# Dyspnea in pregnancy might be related to the incomplete physiological adaptation of the heart

**DOI:** 10.34172/jcvtr.2022.30539

**Published:** 2022-11-26

**Authors:** Atoosa Mostafavi, Mona Feizian, Seyedeh Zahra Fotook Kiaei, Seyed Abdolhussein Tabatabaei

**Affiliations:** ^1^Department of Cardiology, Shariati Hospital, Tehran University of Medical Sciences, Tehran, Iran; ^2^Advanced Thoracic research center, Tehran University of Medical Sciences, Shariati Hospital, Tehran, Iran

**Keywords:** Pregnancy, Dyspnea, Physiologic, Shortness of Breath

## Abstract

***Introduction:*** Dyspnea is a common complaint in pregnant women with no cardiac and pulmonary diseases. We aimed to assess whether physiological dyspnea of pregnancy was correlated with subtle changes in ventricular systolic and diastolic function.

***Methods:*** This cross-sectional study enrolled 40 healthy pregnant women in their second and third trimesters with no complaints of dyspnea and 40 healthy pregnant women in the same trimesters with a complaint of dyspnea. Parameters of echocardiography were compared between the 2 groups.

***Results:*** Global left ventricular ejection fraction (59.65±6.44 and 58.49±4.95 *P*=0.418 in patients without and with dyspnea respectively), and global longitudinal strain were not significantly different (18.72±2.90 and 18.94±3.07, *P*=0.57 in the same order). Global circumferential strain (GCS) was lower in patients with dyspnea. (20.19±4.86 vs 22.61±4.69, *P*=0.03). Systolic volume (33.17±8.94 vs 32.63±8.09) and diastolic volume (80.75±18.73 vs 78.37±16.63) and left ventricular end-diastolic diameter (47.5±4.24 vs 46.23±3.21) were not different (*P*=0.784, 0.560 and 0.146 respectively). Left ventricular end-systolic diameter was significantly lower in the case group (32.52±4.66 vs 29.92±4.05, *P*=0.011). Left atrial area index in the patients with dyspnea was lower(8.13±1.42 vs 8.94±1.4, *P*=0.014). Other findings were a high E/E’ and high pulmonary artery pressure in the patients with dyspnea.

***Conclusion:*** Dyspnea in pregnant women can be a consequence of incomplete physiological adaptation to volume overload in pregnancy. Lower systolic and diastolic diameters of the left ventricle, left atrial area, and left atrial index may lead to increased filling pressure, manifested by a higher E/E’ ratio and pulmonary artery pressure.

## Introduction

 Dyspnea is a common symptom in normal, uncomplicated pregnancies. It is reported in 60% to 70% of normal pregnant women in the third trimester.^1-2^ While dyspnea is insignificant and clinically negligible in some cases, it might be severe and debilitating in a cluster of patients. Only 3.2% of women experience severe dyspnea within their first 20 weeks of gestation, with the rate rising to about 37.5% in the third trimester.^3^ Dyspnea can cause a diagnostic dilemma in pregnant women with previous known cardiac diseases. Multiple causes have been suggested for physiological dyspnea in pregnancy, including anemia, the growing uterus pushing upward on the lungs, increased pulmonary blood volume, and nasal congestion.^4^

 In normal pregnancies, many physiological and hemodynamic cardiovascular alterations occur for adaptation to transient load changes. In the sixth gestational week, plasma volume rises by about 45%, and the red blood cell mass increases by between 20% and 30%.^5^ Cardiac output increases secondary to a higher stroke volume. At the beginning of the second trimester, cardiac output increases by 30% to 50% because of an elevation in stroke volume and pulse rate, leading to volume overload.^6^ Drops in arterial pressure and systemic vascular resistance are features of normal pregnancies and are secondary to the dilatation of the peripheral vessels due to the high levels of progesterone, prostaglandins, and nitric oxide. In addition, the renin-angiotensin- aldosterone system is activated, likely in response to the dilatation of the peripheral vessels and lower blood pressure, and it can cause an increase in vascular tone.^7^ In addition to increased cardiac preload, reduced cardiac afterload and increased left ventricular mass and ejection fraction have also been reported in pregnant women.^8^ Globally, cardiovascular diseases complicate 1% to 4% of pregnancies^9-10^ and are the main cause of mortality during pregnancy.^11^ Some cardiovascular diseases such as congenital cardiac diseases, pulmonary hypertension, and ventricular dysfunction can impact the prognosis of mothers and their neonates.^9^ Thus, understanding the normal remodeling of the maternal heart allows physicians to diagnose and manage abnormal cardiac conditions in early phases. In pregnant women, dyspnea in daily activities can be a symptom of cardiac or pulmonary diseases. Tara et al^12^ assessed 50 pregnant women with dyspnea and reported that 12% of the cases had pulmonary hypertension and 54% suffered from valvular heart diseases, including mitral valve prolapse and regurgitation. Interestingly, a significant correlation was found between the New York Heart Association functional class and the presence of cardiac valvular diseases or pulmonary hypertension. Changes in the physiology of the cardiovascular system in pregnancy and the risks in the course of pregnancy warrant a different and specific approach to the evaluation and management of cardiovascular conditions. Recent advances have enabled pregnant women with cardiac problems to maintain their pregnancy via close, continuous monitoring. It is, therefore, crucial to precisely evaluate ventricular systolic and diastolic function via echocardiography and measure cardiac dimensions and intracardiac pressures in cases of dyspnea. In the present study, we sought to evaluate changes in left ventricular function in terms of more novel parameters, including left ventricular strain, in addition to other functional parameters of ventricular systolic and diastolic functions, in pregnant women suffering from dyspnea with no known or overt causes of dyspnea and compare them with the same parameters in pregnant women without dyspnea.

## Matherials and Methods

 This case-control study was conducted in our hospital between 2020 and 2021. The study population consisted of pregnant women with and without dyspnea with a gestational age of 13 weeks or more who presented to the obstetric/gynecology clinic for routine examinations. Forty pregnant women without dyspnea and 40 pregnant women with dyspnea of New York Heart Association functional class II or higher were selected via convenience sampling. The inclusion criteria were pregnant women with dyspnea as the case group and those without dyspnea as the control group, who were willing to participate in the study, had no previous history of cardiac and respiratory diseases, and had normal baseline echocardiography and spirometry. The exclusion criteria were composed of history of pulmonary diseases, respiratory infections during pregnancy, anemia, history of chronic conditions (eg, renal, hepatic, and rheumatological diseases), history of the consumption of medications affecting the respiratory system (eg, bronchodilators), history of underlying cardiac diseases, structural and congenital abnormalities, and cardiomyopathy at first evaluation. Baseline variables, including age, gestational age, parity, height, weight, body mass index, and medical and drug history, were collected. Dyspnea during pregnancy was defined as a feeling of breath shortness or disturbance in normal breathing that was started during pregnancy and was not caused by new or underlying cardiac or pulmonary diseases. Initial evaluation was performed by physical examination to detect any abnormality in the heart and respiratory systems. The subject’s height, weight, and waist circumference were also measured to exclude the effects of obesity and progressive uterine distention on lung volume. Additionally, routine laboratory tests (cell blood count, blood urea nitrogen and creatinine, liver and thyroid function test, urine analysis and cultures)were done to detect any abnormality. All eligible women underwent transthoracic echocardiography, tissue Doppler ultrasonography, and strain analysis with a Phillips Epic 7 device by an expert echocardiographer who was blind to complaint of dyspnea by the patients. The following variables were collected via echocardiography: left ventricular end- diastolic diameter and volume, left ventricular end-systolic diameter and volume, left ventricular ejection fraction, early and late diastolic mitral inflow velocities (E and A), E/E’ ratio, global circumferential strain (GCS) and global longitudinal strain (GLS), right ventricular end-diastolic diameter and right ventricular systolic calculation via right ventricular peak systolic myocardial velocity (Sm), and tricuspid annular systolic excursion. Finally, left atrial area and pulmonary systolic pressure were calculated, and all the collected data were compared between the 2 study groups.

 Left ventricular end-diastolic volume, left ventricular end-systolic diameter, left atrial area, and right ventricular end-diastolic diameter were measured by simple 2D echocardiography. E-wave, A-wave, and right ventricular Sm velocities, as well as E/E’, were measured by Doppler echocardiography. In addition, GLS and GCS were measured by automated cardiac motion quantification. The measurement of GLS and GCS was done by using 6 gray scale images that were stored on digital media at the time of the collection of other data with care to avoid foreshortening.

**Figure 1:**
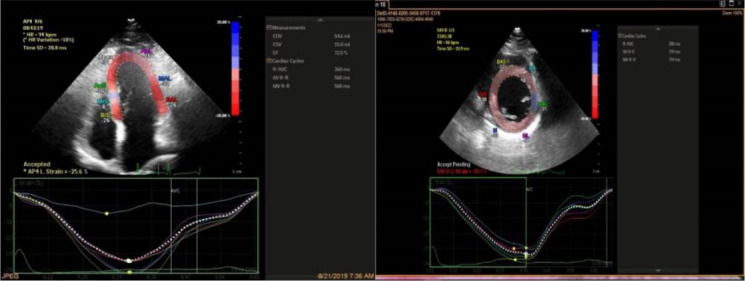
Detection of the endocardial and epicardial border throughout the cardiac cycle (Left: Apical 4 chamber view for global longitudinal strain calculation, right: Parasternal mid ventricular short axis view for global circumferential strain calculation)

**Figure 2:**
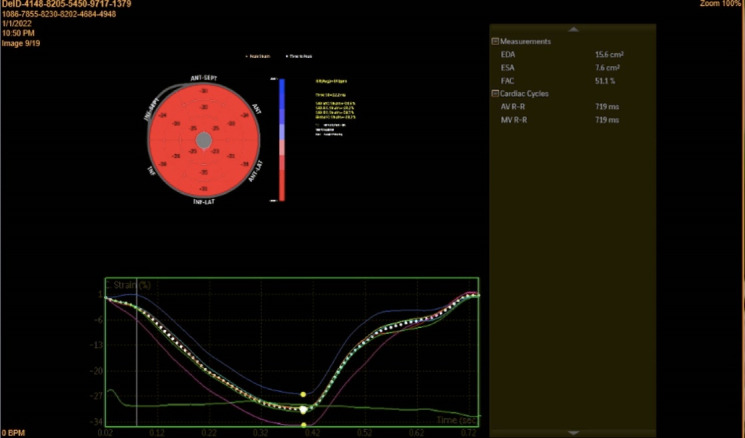
Both strain curve and color coded 17 segments bull’s eye plot.

 Offline analysis was performed, and strain data were generated. Images used for GLS measurement were obtained in 2-, 3-, and 4- chamber views, and images utilized for GCS calculation were acquired in 3 short-axis views at the basal, mid, and apical levels of the left ventricle (Figure 1). The gray- scale rate was kept between 30 and 70 frames per second, and electrocardiography was gated. Automated border detection by software underwent subsequent manual correction and resulting calculated GLS and GCS expressed as the bull’s eye (Figure 2).

 Data collection was done by a single skilled echocardiographer. Inter and intraobserver variabilities were tested via random repetitions by the same echocardiographer at 1-month intervals and by a second echocardiographer, who was blind to the first observer’s measurement. Finally, the parameters were compared between subjects with and without dyspnea. The study protocol was approved by the Review Board of cardiology. (IR.TUMS.MEDICINE.REC.1399.977)

 Statistical analysis was performed with SPSS, version 23.0. For descriptive analysis, the mean and the standard deviation were reported for quantitative parameters. Frequencies and percentages were used for qualitative variables. For the comparison of parametric and nonparametric variables, the t test and the Mann–Whitney U test were employed, respectively. Further, the χ2 test was drawn upon to compare qualitative variables. A *P* value of less than 0.05 was considered statistically significant.

## Results

 Forty pregnant women with dyspnea and 40 pregnant women without dyspnea were analyzed in our study. The mean age of the subjects with and without dyspnea was 30.57 ± 5.75 and 30.52 ± 4.71 years, respectively (*P* = 0.965). The mean gestational age was 25.81 ± 7.81 and 29.73 ± 5.67 weeks, respectively, which was significantly higher in the patients without dyspnea (*P* = 0.015). There were no significant differences in weight, body mass index, and waist circumference between the 2 groups. The entire study population’s baseline characteristics are summarized in Table 1.

**Table 1:** Baseline characteristics of subjects with and without dyspnea

**Parameter**	**Subjects without dyspnea**	**Subjects with dyspnea**	***P*** ** value**
Age (years)	30.52 ± 4.71	30.57 ± 5.75	0.965
Weight (kg)	77.6 ± 13.07	81.01 ± 10.04	0.207
WC (mm)	105.89 ± 11.86	107.21 ± 6.17	0.546
Height (cm)	160.44 ± 2.99	160.02 ± 3.07	0.547
Gestational age (weeks)	29.73 ± 5.67	25.81 ± 7.81	0.015
Body mass index (kg/m^2^)	30.12 ± 4.76	31.59 ± 3.4	0.125
Gravidity (mean)	1.93	1.89	0.235
Parity	0.66	0.73	0.540

Abbreviations: WC, waist circumference.

 Evaluation of echocardiographic parameters showed that while global left ventricular ejection fraction and GLS were not significantly different between the 2 groups (P = 0.418 and P = 0.575, respectively), GCS was lower in the patients with dyspnea, compatible with mild left ventricular systolic dysfunction (*P* = 0.03). Systolic and diastolic volume and left ventricular end-diastolic diameter were not different between the 2 groups, whereas left ventricular end-systolic diameter was significantly lower in the case group (*P* = 011). Interestingly, left atrial area and left atrial volume index in the patients with dyspnea were lower than those in the control group (*P* = 0.018 and *P* = 0.014, respectively). Although relative wall thickness was within the normal range in both groups, the women with dyspnea showed a trend for higher relative wall thickness (*P* = 0.08). Left ventricular mass and left ventricular mass index were not significantly different between the 2 groups (*P* = 0.747 and *P* = 0.295, respectively). Other scintillating findings were a high E/E’ and a high pulmonary artery pressure in the patients with dyspnea compared with the control group (*P* = 0.024 and *P* = 0.008, respectively). to detect by routine echocardiography; nonetheless, it causes symptoms of dyspnea in pregnant women. A lower GCS is another finding that should be considered as a cause of dyspnea in pregnancy. The study population’s echocardiographic variables are presented in detail in Table 2.

**Table 2:** Echocardiographic parameters in subjects with and without dyspnea

**Parameter**	**Subjects without dyspnea**	**Subjects with dyspnea**	***P*** ** value**
LA area (cm^2^)	15.88 ± 2.68	14.52 ± 2.18	0.018
RWT (mm)	0.34 ± 0.04	0.37 ± 0.05	0.008
LVEDD (mm)	47.5 ± 4.24	46.23 ± 3.21	0.146
LVESD (mm)	32.52 ± 4.66	29.92 ± 4.05	0.011
LVEDV (mL)	80.75 ± 18.73	78.37 ± 16.63	0.560
LVESV (mL)	33.17 ± 8.94	32.63 ± 8.09	0.784
EF (%)	59.65 ± 6.44	58.49 ± 4.95	0.418
GLS (%)	18.72 ± 2.90	18.94 ± 3.07	0.575
GCS (%)	22.61 ± 4.69	20.19 ± 4.86	0.03
RVD (mm)	23.11 ± 2.70	22.68 ± 2.55	0.477
TAPSE (mm)	25.39 ± 3.59	24.76 ± 3.75	0.456
RVsm (cm/s)	13.4 ± 1.71	13.62 ± 1.85	0.595
PAP (mm Hg)	23.47 ± 3.35	25.26 ± 2.28	0.008
LA size (mm)	30.31 ± 3.46	29.31 ± 2.94	0.179
LA area index (cm^2^/m^2^)	8.94 ± 1.4	8.13 ± 1.42	0.014
LV mass (gr)	128.31 ± 26.33	126.52 ± 21.62	0.747
LV mass index (gr/m^2^)	70.13 ± 14.51	67 ± 11.14	0.295
LV Diastolic volume index (mL/m^2^)	45.17 ± 8.98	43.64 ± 10.46	0.497
LV Systolic volume index (mL/m^2^)	18.34 ± 4.26	18 ± 4.61	0.737
A wave velocity (cm/s)	67.15 ± 18.95	72.96 ± 20.20	0.20
E wave velocity (cm/s)	76.42 ± 14.54	82.72 ± 17.16	0.088
E' wave velocity (cm/s)	9.17 ± 1.31	9.47 ± 2.10	0.466
E/E' waves ratio	8 ± 1.17	9.13 ± 2.76	0.024
E/A wave ratio	1.19 ± 0.33	1.20 ± 0.41	0.911

Abbreviations: EF, ejection fraction; LA, left atrium; LV, left ventricle; LVEDD, left ventricular end diastolic diameter; LVEDV, left ventricular end diastolic volume; LVESD, left ventricular end systolic diameter; LVESV, left ventricular end systolic volume; RVD, RV diastolic diameter; RWT, relative wall thickness; RVsm, right ventricular systolic motion velocity; TAPSE, tricuspid annular plane systolic excursion.

## Discussion

 During a healthy pregnancy, pulmonary and cardiac systems are affected by both mechanical and biochemical pathways. Alterations in the hormonal pathway are deemed the principal reason for ventilatory changes in respiratory function.^13^ The mechanical effects of uterine distension and secondary elevation of the diaphragm also cause reduced lung volume and altered chest walls. Despite a lower lung volume in women with a healthy pregnancy, spirometry remains within the normal limit. Cardiac function, cardiac chambers, and the circulatory system are also affected in a normal pregnancy, and blood volume increases from 30% to 50%. Other major cardiovascular changes associated with a normal pregnancy include increased stroke volume, cardiac output, heart rate, and venous return, as well as a paradoxical fall in systemic vascular resistance and blood pressure.^14^ These hemodynamic alterations are mandatory to protect the mother from the consequences of hemorrhage and for the optimal growth of the fetus.^4^ The other alteration in the maternal cardiopulmonary system is the effect of pregnancy on pulmonary artery pressure and left ventricular end-diastolic pressure. Sharma et al^15^ observed that compared with the pre- pregnancy period, a rise in left ventricular end-diastolic pressure and systolic pulmonary artery pressure would occur in pregnancy. It is well known that a diminished lung volume, along with increased pulmonary artery pressure, left ventricular volume, and end-diastolic pressure, can cause pulmonary congestion and dyspnea. As a result of these changes, dyspnea is reported in 70% of healthy pregnant women beginning from the first trimester and continuing throughout the pregnancy.^13^ Be that as it may, it is recommended that pregnant women with dyspnea be visited by a cardiologist and undergo echocardiography for the evaluation of other pathological causes of dyspnea as the mentioned physiological adaptations may exacerbate preexisting cardiac problems and pregnancy related cardiomyopathy.

 Heart chambers size and left ventricular function are altered in a normal pregnancy. All 4 cardiac chambers enlarge significantly compared with those in nonpregnant women. Left ventricular mass increases as well. Moreover, left ventricular systolic function undergoes different alterations in all trimesters, and the results are contradictory in different studies. Tso et al^16^ showed that left ventricular ejection fraction was significantly higher in the second trimester but it fell in the third trimester. In contrast, Estensen et al^17^ reported that left ventricular contractility was significantly lower in pregnancy compared with 6 months postpartum. Identifying and understanding the structure and function of the maternal heart are clinically essential for cardiovascular management. Recently, speckle-tracking echocardiography and evaluation of ventricular strain, particularly GLS and GCS, have provided more comprehensive information regarding ventricular dysfunction in pregnant women with dyspnea and subclinical abnormalities.^14^

 The results of the current study revealed that mean GCS in pregnant women with dyspnea decreased significantly compared with healthy pregnant women, while changes in GLS index were not significant and this subtle abnormality of LV function which is not still evident in other parameters as GLS might be the cause of dyspnea in pregnant women who appears normal in 2-dimensional echocardiography. Assessment of the other structural and functional parameters of the left ventricle in pregnant women with dyspnea showed significantly larger relative wall thickness, E/e’ ratio, and pulmonary artery pressure and significantly lower left ventricular end-systolic diameter, left atrial area, and left atrial area index by comparison with the control group. In addition, left ventricular end-diastolic diameter and left atrial size were lower in the group with dyspnea than in the controls, but this difference was not statistically significant. Our results are in line with some previous reports. Goland et al^18^ evaluated dyspnea associated with cardiac ventricular function during pregnancy by Doppler echocardiography. They reported that left ventricular wall thickness in women with and without dyspnea was 10.1 ± 1.1 mm and 8.9 ± 0.9 mm, respectively. Additionally, deceleration time was decreased, and pulmonary artery pressure was significantly elevated in the pregnant women with dyspnea than in those without dyspnea (26.8 ± 6.2 mm vs 19.0 ± 6.5 mm Hg). Goland and colleagues also reported that left ventricular end-systolic diameter was lower in their patients with dyspnea than in their controls, but this decrease failed to constitute statistical significance.

 Physiologically, the enlargement of cardiac chambers is expected due to an elevated blood volume in pregnancy. Nevertheless, we found that although cardiac chamber size in both groups was within the normal range, the group with dyspnea had a significantly lower chamber size than the group without dyspnea. On the other hand, a significant rise in the ventricular diastolic index of E/E’ in pregnant women with dyspnea can reflect degrees of myocardial stiffness and higher left ventricular end-diastolic pressure. Hence, we conclude that the increase in cardiac chamber size is a physiological adaptation to maintain left ventricular end- diastolic pressure within the normal range and in some pregnant women, this adaptation is not enough, leading to physiological dyspnea of pregnancy.

 Some other studies have also reported a reduction in ventricular diameters among pregnant women with dyspnea. Barut et al,^4^ having evaluated clinical and echocardiographic parameters in pregnant women with dyspnea, reported a significant increase in left ventricular end-diastolic diameter and pulmonary artery pressure and a decrease in left ventricular end-systolic diameter in the presence of dyspnea compared with controls.

 Another theory for the justification of physiological dyspnea in pregnancy is a subtle reduction in left ventricular function that is not detectable with a simple assessment of ejection fraction. The measurement of cardiac strain values by speckle-tracking echocardiography can provide precise information on regional and global disturbances in the function of ventricular contractions. These strain values are measured in longitudinal and circumferential dimensions.^19^ Among these strain values, GCS index is a sensitive parameter for regional myocardial function. Tamrat et al^20^ reported a significant fall in GCS among pregnant women with peripartum cardiomyopathy in spite of normal ejection fraction and left ventricular end-diastolic diameter. Additionally, GLS exhibited a drop in comparison with controls, but this reduction was nonsignificant. A reduction in left ventricular strain parameters, consisting of GLS, GCS, and GRS(Global radial strain), in pregnant women with gestational hypertension and severe or early preeclampsia has been reported.^21^ Sugahara et al^22^ reported significant decreases in ejection fraction, GLS, and GCS and increases in left ventricular systolic and diastolic diameters among pregnant women with peripartum cardiomyopathy. Another study also evaluated cardiac abnormalities in different pregnancy trimesters and compared the findings with those in nonpregnant women.

 The results revealed that with advances in gestational age, ejection fraction, GLS, and GCS decreased, while left ventricular systolic and diastolic diameters and relative wall thickness index increased.^23^ There are only a few studies on the association between changes in cardiac parameters and dyspnea in pregnancy, and our results are consistent with their findings.^2-18^

 A lower left ventricular end-systolic diameter, a lower left atrial area, a higher E/E’, and a higher pulmonary artery pressure make this hypothesis probable that physiological adaptation through an increase in the size of the heart chambers is not enough in some pregnant women to meet the higher blood volume of pregnancy and ultimately leads to a higher left ventricular end-diastolic pressure and a higher pulmonary artery pressure. The elevation in left ventricular end-diastolic pressure and pulmonary artery pressure is too subtle

 The limitation of our study is its relatively small sample size. Larger study populations with the application of spirometry may yield more comprehensive results.

 Another limitation of our study is the use of NYHA FC for assessing the presence of dyspnea as opposed to scales such as Dyspnea VAS etc.

## Conclusion

 Dyspnea in pregnant women can be a consequence of incomplete physiological adaptation to volume overload in pregnancy. Lower systolic and diastolic diameters of the left ventricle, left atrial area, and left atrial index may lead to increased filling pressure, manifested by a higher E/E’ ratio and pulmonary artery pressure. However, subtle systolic dysfunction, indicated by a low GCS value, should be considered as well.

## Author Contributions

**Conceptualization:** Atoosa Mostafavi, Seyed Abdolhussein Tabatabaei.

**Methodology:** Atoosa Mostafavi, Seyed Abdolhussein Tabatabaei, Seyede Zahra Fatook Kiaei.

**Validation:** Atoosa Mostafavi, Seyed Abdolhussein Tabatabaei, Seyede Zahra Fatook Kiaei.

**Formal Analysis:** Seyede Zahra Fatook Kiaei, Atoosa Mostafavi, Seyed Abdolhussein Tabatabaei, Mona Feizian.

**Investigation:** Seyede Zahra Fatook kiaei, Atoosa Mostafavi, Seyed Abdolhussein Tabatabaei, Mona Feizian.

**Resources:** Seyede Zahra Fatook Kiaei, Atoosa Mostafavi, Seyed Abdolhussein Tabatabaei, Mona Feizian.

**Data Curation:** Seyede Zahra Fatook Kiaei, Atoosa Mostafavi, Seyed Abdolhussein Tabatabaei, Mona Feizian.

**Writing—Original Draft Preparation:** Mona Feizian, Atoosa Mostafavi.

**Visualization:** Atoosa Mostafavi.

**Supervision:** Atoosa Mostafavi.

**Project Administration:** Atoosa Mostafavi, Seyed Abdolhussein Tabatabae.

**Funding Acquisition:** Atoosa Mostafavi, Seyed Abdolhussein Tabatabae.

## Funding

 The authors declare that there are no funding supports.

## Ethical Approval

 The study protocol was approved by the Review Board of cardiology. (IR.TUMS.MEDICINE.REC.1399.977)

## Compething Interests

 The authors declare that there are no conflicts of interest regarding the publication of this article.

## References

[1] Gilbert R, Auchincloss JH Jr (1966). Dyspnea of pregnancy Clinical and physiological observations. Am J Med Sci.

[2] Reeder CF, Hambright AA, Fortner KB (2018). Dyspnea in pregnancy: a case report of a third trimester mediastinal mass in pregnancy. Am J Case Rep.

[3] Choi HS, Han SS, Choi HA, Kim HS, Lee CG, Kim YY (2001). Dyspnea and palpitation during pregnancy. Korean J Intern Med.

[4] Barut MU, Güngören F, Kaçmaz C (2019). Assessment of clinical and echocardiographic findings of pregnant women with dyspnea. Med Sci Monit.

[5] Chapman AB, Abraham WT, Zamudio S, Coffin C, Merouani A, Young D (1998). Temporal relationships between hormonal and hemodynamic changes in early human pregnancy. Kidney Int.

[6] Carbillon L, Uzan M, Uzan S (2000). Pregnancy, vascular tone, and maternal hemodynamics: a crucial adaptation. ObstetGynecolSurv.

[7] Savu O, Jurcuţ R, Giuşcă S, van Mieghem T, Gussi I, Popescu BA (2012). Morphological and functional adaptation of the maternal heart during pregnancy. Circ Cardiovasc Imaging.

[8] Valensise H, Novelli GP, Vasapollo B, Di Ruzza G, Romanini ME, Marchei M (2001). Maternal diastolic dysfunction and left ventricular geometry in gestational hypertension. Hypertension.

[9] Regitz-Zagrosek V, Roos-Hesselink JW, Bauersachs J, Blomström-Lundqvist C, Cífková R, De Bonis M (2018). 2018 ESC Guidelines for the management of cardiovascular diseases during pregnancy. Eur Heart J.

[10] Emmanuel Y, Thorne SA (2015). Heart disease in pregnancy. Best Pract Res Clin ObstetGynaecol.

[11] Siu SC, Sermer M, Colman JM, Alvarez AN, Mercier LA, Morton BC (2001). Prospective multicenter study of pregnancy outcomes in women with heart disease. Circulation.

[12] Tara F, Vakilian F, Moosavi-Baigy F, Salehi M, Moghiman T (2015). Prenatal and cardiovascular outcome in pregnant patients with dyspnea. Res Cardiovasc Med.

[13] LoMauro A, Aliverti A (2015). Respiratory physiology of pregnancy: physiology masterclass. Breathe (Sheff).

[14] Somani SS, Sunandini R, Somani SG (2016). Role of echocardiography for assessment of cardiovascular haemodynamics during pregnancy. Int J Reprod Contracept Obstet Gynecol.

[15] Sharma R, Kumar A, Aneja GK (2016). Serial changes in pulmonary hemodynamics during pregnancy: a non-invasive study using doppler echocardiography. Cardiol Res.

[16] Tso GJ, Lee JM, Shaban NM, Lui GK, Trivedi HA, Cohen MN (2014). Normal echocardiographic measurements in uncomplicated pregnancy, a single center experience. J Cardiovasc Dis Res.

[17] Estensen ME, Beitnes JO, Grindheim G, Aaberge L, Smiseth OA, Henriksen T (2013). Altered maternal left ventricular contractility and function during normal pregnancy. Ultrasound Obstet Gynecol.

[18] Goland S, Perelman S, Asalih N, Shimoni S, Walfish O, Hallak M (2015). Shortness of breath during pregnancy: could a cardiac factor be involved?. Clin cardiol.

[19] Zhang K, Sheu R, Zimmerman NM, Alfirevic A, Sale S, Gillinov AM (2019). A comparison of global longitudinal, circumferential, and radial strain to predict outcomes after cardiac surgery. J CardiothoracVascAnesth.

[20] Tamrat R, Kang Y, Scherrer-Crosbie M, Levine LD, Arany Z, Lewey J (2021). Women with peripartum cardiomyopathy have normal ejection fraction, but abnormal systolic strain, during pregnancy. ESC Heart Fail.

[21] Moors S, van Oostrum NHM, Rabotti C, Long X, Westerhuis M, Kemps HMC (2020). Speckle tracking echocardiography in hypertensive pregnancy disorders: a systematic review. ObstetGynecolSurv.

[22] Sugahara M, Kagiyama N, Hasselberg NE, Blauwet LA, Briller J, Cooper L (2019). Global left ventricular strain at presentation is associated with subsequent recovery in patients with peripartum cardiomyopathy. J Am Soc Echocardiogr.

[23] Cong J, Fan T, Yang X, Squires JW, Cheng G, Zhang L (2015). Structural and functional changes in maternal left ventricle during pregnancy: a three-dimensional speckle-tracking echocardiography study. Cardiovasc Ultrasound.

